# Age, Sex, and Profession Difference Among Health Care Workers With Burnout and Metabolic Syndrome in Taiwan Tertiary Hospital—A Cross-Section Study

**DOI:** 10.3389/fmed.2022.854403

**Published:** 2022-04-14

**Authors:** Huel-Ju Tsai, Meng-Ting Tsou

**Affiliations:** ^1^Departments of Health Evaluation Center, MacKay Memorial Hospital, Taipei City, Taiwan; ^2^Department of Family Medicine, MacKay Memorial Hospital, Taipei City, Taiwan; ^3^Department of Occupation Medicine, MacKay Memorial Hospital, Taipei City, Taiwan; ^4^Department of MacKay Junior College of Medicine, Nursing, and Management, New Taipei City, Taiwan

**Keywords:** health care workers, burnout, metabolic syndrome, Taiwan, sex

## Abstract

**Background:**

This cross-sectional study aimed to analyze the association between burnout, work-related factors and metabolic syndrome (MetS) among various health-care workers (HCWs) at a tertiary hospital in Taiwan.

**Methods:**

Relevant demographic data were obtained through written questionnaires. Information about psychosocial and work conditions, including assigned department, working hours, shifts, and sleep condition, was obtained. Burnout was evaluated according to the Chinese version of Maslach Burnout Inventory–Health Services Survey. MetS was analyzed according to the criteria of the National Cholesterol Education Program of Taiwan–Treatment Panel for Adults III.

**Results:**

A total of 1,055 non-doctor/nurse and 2,078 doctor/nurse staff with a median age of 45.2 and 36.1 years participated in this study. The incidence of burnout was nearly 6.42 and 6.68% and that of MetS was 31.4 and 13.5% in non-doctor/nurse and doctor/nurse staff, respectively. The results showed that burnout induced a higher Odds ratio (OR) of MetS in the doctor/nurse group (OR = 1.96, *p* = 0.01). Other factors, such as night shift and seniority (>10 years), led to a higher OR of MetS, but a decreased risk was observed based on seniority (2–4 years). Further, young female participants and young doctors/nurses with burnout had a higher OR of MetS compare to other groups (OR = 2.43 and 2.32, *p* < 0.05).

**Conclusion:**

The study results suggested positive relationship between burnout and MetS in young female staffs and young doctors/nurses. For doctor/nurse staff with higher seniority or more night shifts, strategies are needed to prevent burnout and MetS.

## Introduction

Health care workers (HCWs) need to respond immediately to patients and families, making their jobs stressful (1). HCWs are at risk for adverse health effects, including metabolic syndrome (MetS) and burnout, due to heavy workloads, patient cases, long service hours, and shift work (1–4).

According to Schaufeli et al., health professionals, police officers, and teachers have a high risk of burnout (5). If their relationship with patients/clients remains constant and intense, increased care requirements is associated with a higher risk of burnout (5). In a study conducted at a regional hospital in Taiwan, Chou et al. observed that among the five professions in the medical field, the highest prevalence of work-related stress occurred in nurses (66%), followed by medical assistants (61.8%), doctors (38.6%), administrative staff (36.1%), and medical technicians (31.9%) (2).

The association between age and burnout is not frequently explored in studies. Akkuş et al. analyzed the association between age and burnout and observed a decrease in the scores of the three domains of burnout with increasing age (6). In a meta-analysis, Gómez-Urquiza et al. observed that the age of young individuals was a significant factor in the emotional exhaustion and depersonalization domains of burnout (7).

In a study that analyzed a sample of the adult working population in Finland, Ahola et al. found that women with burnout had a U-shaped distribution. A negative association was observed between age and burnout in the first years of work (age: 18–40 years) and around the age of 60–64 years. In young women, a higher prevalence of burnout was found among younger workers (8).

In studies conducted by Chico-Barba et al. and Merces et al., MetS was prevalent in 38.7% Mexican nurses (9) and 24.4% primary care nursing professionals in Brazil, respectively (10). A study conducted in a tertiary hospital in Taiwan showed that the highest prevalence of MetS was observed in physicians (18.3%), followed by administrative staff (15.4%), medical technicians (9.4%), and nurses (6.6%) (3).

According to a previous study that reported the association between burnout and MetS among nursing staff working in Brazil primary health-care clinics, burnout increases the risk of MetS by 1.45% (95% confidence interval [CI] = 1.17–1.81) (10). Another study conducted on nursing staff working in tertiary hospital in Taiwan showed that those with burnout had a higher risk of MetS (odds ratio [OR] = 1.70, 95% CI = 1.04–3.05) (11). A study by Chico-Barba et al. observed an association between increased abdominal circumference and domains of emotional exhaustion burnout (adjusted OR [aOR] = 14.95; 95% CI = 1.5–148.7] and night shift (aOR = 12.39; 95% CI = 1.02–150.5) (9). However, few studies have explored the association between burnout, work factors, and professional categories and MetS among staff at a tertiary hospital in Taiwan.

In addition, age and sex have always been adjusted factors; however, in real-world setting, there are different proportions of hospital staff, including doctor/nurse and non-doctor/nurse groups, male and female groups and different age groups. Earlier studies have reported that age and profession affect burnout and MetS, respectively (6–10). Therefore, our hypothesis mainly included exploring the impact of burnout on MetS according to different age groups, sex and professions. This study aimed to determine the specific group in which burnout increases the risk of MetS. For these HCWs, in addition to traditional lifestyle modification education, this study provided evidence on preventive policies for burnout and the required work-related factors.

## Materials and Methods

### Study Design and Participants

This cross-sectional study was conducted at the Health Evaluation Center, a 2,000-bed tertiary hospital in Northern Taiwan. MetS data were obtained from yearly routine physical examinations by HCWs.

Questionnaires were collected between December 2018 and March 2019 during employee physical examination. The contents of the self-administered questionnaire were explained to participants face to face before they were administered the questionnaire. If more than one-fourth of the questionnaire was not filled out, the questionnaire was excluded. Workforce in all departments, such as doctors, nurses, technicians, administrative staff, pharmacists, radiologists, and nutritionists, were included. Since doctors and nurses encounter the same conditions such as facing patients' problems on time, taking care of the patients, work patterns, and shift patterns, they were categorized into the doctor/nurse group and the remaining staff was categorized into the non-doctor/nurse for analysis. A total of 3,288 doctors/nurses and 1,943 non-doctors/nurses were invited to participate, and 2,078 and 1,055 of them, respectively, completed the questionnaire (actual response rate was 63.2 and 54.3%, respectively). In addition, a sample size of 2,533 achieved 90% power using the two tailed test (OR = 1.59, probability of null hypothesis = 0.15, alpha error = 0.05, power = 0.9, and R^2^ for other confounding factors = 0.5) (12). In total of 3,133 participants were included in this study, implying a sufficient statistical power.

### Sociodemographic Data and Working Information

Information was obtained through structured questionnaires, designed according to the Institute of Labor, Occupational Safety, and Health Ministry of Labor (13). In this survey, analysis of the internal consistency reliability showed the Cronbach α to be between 0.82 to 0.93, having good reliability. The questionnaire included: basic data and socioeconomic status (included age, education level, department, seniority, working hours, work style, Cronbach α: 0.93); personal lifestyle (included sleep duration, smoking, alcohol, exercise, fruit and vegetable intake, Cronbach α: 0.89); psychosocial work environment (Maslach Burnout Inventory–Health Services Survey test; MBI-HSS, Cronbach α: 0.82); mental health status (Brief Symptom Rating Scale-5, BSRS-5, Cronbach α: 0.90) (13).

### Anthropometric Measurements

Data on following baseline characteristics were collected, age, body height, body weight, waist circumference (WC), and blood pressure (BP). Height was measured with a standard stadiometer with an approximation value of 0.01 cm. Weight was measured using a set of calibrated electronic scales. The participants wore light clothes weighing ~0.01 kg. WC was measured at the anatomical region of the midpoint between the lower rib and the upper point of the iliac crest at the end of expiration. After the blood was drawn, rest for 10 min, and then start to use the digital electronic sphygmomanometer (MORON HEM7156, Japan) to measure the two arms. The arm with the higher blood pressure was taken as the measuring arm, and the first value was taken. Then, after a 10 min rest, measure the second time again, and average the two times to the final measured value (14). Height, weight, WC, and BP measurements were performed and recorded by trained nurses working at the Health Evaluation Center who were blinded to the patients' information.

### Personal Lifestyle

Information concerning personal habits, including smoking status (yes/no: current or past/never), alcohol consumption (yes/no: ≥2 drinks per week/0–1 drink per week), exercise (yes: ≥3 times/week, ≥30 min/set), fruit intake (one serving: equivalent to one medium orange, apple, or guava), and vegetable intake (one serving: equivalent to a 15-cm plate or more than half a bowl), was collected (13). Information regarding the length of sleep duration and self-assessment of sleep quality was obtained through a self-administered questionnaire (13). Participants were asked to self-assess their sleep quality, and the response was classified as either “good” or “poor.”

### Work Characteristics

Study participants were asked to provide information on the following during the past month before the survey: total weekly working hours and work style. The work style was divided into three classes according to the work shift that starts in the morning (D means day class: 8:00–16:00), evening (E means evening class: 16:00–24:00), and night (N means night class: 24:00–8:00). Night shift included the midnight work schedule. Participants were categorized into the following three types of work: regular class (D), night shift (E or N, fixed or rotation), and three shifts (D, E, and N rotation) (13).

### Laboratory Data Acquisition and Analysis

Sample collection and analysis were performed in a standard laboratory with international accreditation (ISO-15189). All participants were informed to start fasting at 10:00 PM the night before and started blood draw at 8:00–10:00 AM the following day. at the Health Evaluation Center. The blood samplings were collected in a BD Vacutainer SST^TM^ (Becton Dickinson, Franklin Lakes, NJ, USA) sample collection tube. Sample collection and analysis principles were based on the standard requirements mentioned in the Clinical and Laboratory Standards Institute guidelines (specimen choice, collection, and handling; approved guideline H18-A3).

A Hitachi 7170 Automatic Analyzer (Hitachi Corp., Hitachinaka Ibaraki, Japan) was used to measure the levels of fasting glucose, lipid markers (including low- and high-density lipoprotein cholesterol [HDL-C: homogeneous enzymatic colorimetric assay], and triglycerides (TG).

### Burnout Assessment

Burnout domains were evaluated using the Chinese version of the MBI-HSS test (15) by Lu et al. (Cronbach's: 0.84) (16). The MBI-HSS evaluates three domains—emotional exhaustion (EE), depersonalization (DP), and personal accomplishment (PA); it included 22 questions. The seven-point Likert scale with scores ranging from 0 (never felt) to 6 (every day), measures the frequency of occurrence. The full scores of EE, DP and PA were 54, 30, and 48, respectively. According to the classification standard of Maslach et al. [15], an EE of score ≥27, a DP score of ≥13, and a PA ≤31 is categorized as high job burnout; if an EE score 17–26, a DP score 7–12, and a PA score 32–38 is considered moderate job burnout; and an EE score of ≤16, a DP score of ≤6, and a PA score of ≥39 is considered low job burnout. In previous studies, the psychological work demands, EE and DP were directly related to burnout; however, a negative association was observed between burnout with PA (15, 16). The scale has good reliability and validity. The internal consistency coefficients (Cronbach's α) of the three dimensions of EE, DP, and PA were 0.77, 0.83, and 0.82, respectively (16).

### Classification According to the Metabolic Syndrome

To analyze the metabolic score, we used the National Cholesterol Education Program–Adult Treatment Panel III (NCEP-ATP III) classification with specific cutoff values for abdominal obesity in the Taiwanese population.

For the standardization of the MetS criteria, individuals should meet at least three of the following five criteria to be included: (1) WC ≥90 cm for men and ≥85 cm for women, (2) HDL-C level <40 mg/dL for men and <50 mg/dL for women, (3) TG level ≥150 mg/dL, (4) BP ≥130/85 mmHg or receiving treatment for hypertension, and (5) fasting blood glucose ≥100 mg/dL or receiving treatment for type 2 diabetes mellitus (17).

### Ethics Approval

The study protocol was reviewed and approved by the Research Ethics Committee on Human Beings of MacKay Memorial Hospital (research project number 18MMHISO150).

All participants provided written informed consent. Confidentiality in data collection was preserved, considering ethical issues, individual autonomy, and respect. This study was conducted in accordance with all guidelines of the Declaration of Helsinki. To ensure data confidentiality, patient identification information was replaced with a sheet number.

### Statistical Analysis

Descriptive analysis was performed to characterize the population sample. Data are presented as mean ± standard deviation for continuous variables and numbers and percentages for categorical variables. According to the cutoff value of the NCEP-ATP III, the MetS factors were classified into two categories. For each factor, frequencies and percentages were calculated.

Student's *t*-test was used to compare continuous variables between groups. For the analysis of categorical variables, the chi-square test was used. Spearman correlation coefficient matrix was used to analyze the relation between seniority with working hours and night shift. Multivariate logistic regression was used to investigate associations between MetS, burnout, and factors associated with work after adjusting for age, sex, educational level, and lifestyle characteristics and determine the association of burnout and prevalence of MetS in the age group stratified by sex and profession.

For statistical analysis, SPSS Statistics version 22 (IBM Corp., Armonk, NY, USA) for Windows was used, and *p* < 0.05 was considered bilateral.

## Results

### Characteristics of the Participants

A total of 2,078 doctors/nurses and 1,055 non-doctors/nurses were included in this study. Table 1 showed the mean age and proportion of male participants was significantly higher in the non-doctor/nurse group than that in the doctor/nurse group (45.24 ± 10.21 vs. 36.14 ± 9.83 years and 19.43% vs. 7.42%, respectively; *p* < 0.001). The educational level in the doctor/nurse group was significantly higher than that in the non-doctor/nurse group. Vegetable and fruit intake, exercise, and smoking behavior tended to be higher in the non-doctor/nurse group than in the doctor/nurse group. The seniority of the doctor/nurse staff was mostly ≤10 years. In contrast, seniority of 41% of the non-doctor/nurse staff was >10 years. The working hour was obviously higher in the doctor/nurse group than in the non-doctor/nurse group. Moreover, 44% of the doctor-nurse staff worked for >46 h per week, and nearly 61% of the doctor/nurse group needed to perform three shifts and night shift. No statistically statistic different was observed between the non-doctor/nurse and doctor/nurse groups in terms of the sleep duration (mean and standard deviation: 6.58 [0.78] vs. 6.60 [1.04] h; *p* = 0.325]. A higher prevalence of poor sleep quality was observed in the doctor/nurse group than that in the non-doctor/nurse group (64.43% vs. 54.78%; *p* < 0.001).

**Table 1:** Demographics, lifestyle factors, work-related characteristics, and sleep condition of the study in this table participants stratified according to profession.

**Variables**	**Non-doctor/nurse**		**Doctor/nurse**		***p*-value**
	**(*****n*** **=** **1,055)**		**(*****n*** **=** **2,078)**		
***Demographic characteristics***					
Age, years (mean, SD)	45.24 (10.21)		36.14 (9.83)		<0.001
Men, *n* (%)	205	19.43	154	7.42	<0.001
Education level, *n* (%)					<0.001
≦Senior high school	343	32.55	44	2.14	
College	568	53.81	1,881	90.52	
≧Graduate School	144	13.64	153	7.34	
***Lifestyle characteristics***					
Smoking, *n* (%) ^a^	55	5.23	57	2.74	0.002
Drink, *n* (%) ^b^	73	6.89	140	6.72	0.745
Exercise, *n* (%) ^c^	648	61.44	950	45.72	<0.001
Fruit intake (serving/day), *n* (%) ^d^					0.001
0	112	10.63	371	17.85	
1	624	59.15	1,179	56.74	
≧2	319	30.22	528	25.41	
Vegetable intake (serving/day), *n* (%) ^e^					0.01
0	43	4.08	87	4.18	
1	342	32.45	878	42.24	
2	457	43.32	798	38.43	
≧3	213	20.15	315	15.15	
***Work Characteristics***					
Seniority, *n* (%)					0.001
<2 years	172	16.35	431	20.73	
2–4 years	183	17.28	318	15.32	
4–10 years	266	25.23	653	31.43	
>10 yeras	434	41.14	676	32.52	
Working hours/week, *n* (%)					<0.001
≦45 h	816	77.30	1,152	55.43	
46–50 h	217	20.64	678	32.61	
51–59 h	16	1.52	148	7.14	
≧60 h	6	0.54	100	4.82	
Work style, *n* (%)					<0.001
Regular class	768	72.79	793	38.14	
Night shift	23	2.14	153	7.34	
Three shifts	264	25.07	1,132	54.52	
***Sleep condition***					
Sleep duration, hours (mean, SD)	6.58 (0.78)		6.60 (1.04)		0.325
Self-assessment of sleep quality, *n* (%)					<0.001
Good	477	45.22	760	36.57	
Poor	578	54.78	1,318	64.43	

a *Smoking status (current or past/never)*.

b *Alcohol consumption (0–1 drinks per week/≧2 drinks per week)*.

c *Exercise (≧3 times/week, ≧30 min/time)*.

d *Fruit intake: One serving: equivalent to one medium orange, apple or guava*.

e *Vegetable intake: One serving: equivalent to 15 cm plate or more than half a bowl*.

*n, numbers; SD, Standard deviation*.

### Prevalence of MetS and MetS Factors, Burnout, and Burnout Domains Stratified According to Profession

Table 2 shows mentioned the prevalence and condition of MetS and burnout of the two groups. The prevalence of MetS and MetS factors in the non-doctor/nurse group was statistical significantly higher than that in the doctor/nurse group (MetS prevalence: total, 19.0%; 29.81% vs. 13.54, *p* < 0.001). In the non-doctor/nurse group, hyperglycemia and central obesity ranked by the prevalence as the first and second, respectively. In the doctor/nurse group, central obesity and high BP ranked by the prevalence as the first and second, respectively. Regarding the prevalence of burnout (burnout prevalence: total, 6.6%; non-doctor/nurse group vs. doctor/nurse group, 6.42% vs. 6.68%; *p* = 0.88), no statistically difference was observed between the groups. However, the analysis of the three domains showed that the score and prevalence of emotional exhaustion and depersonalization were higher in the doctor/nurse group than that in the non-doctor/nurse group.

**Table 2:** Prevalence of MetS, components of MetS, burnout and burnout domains stratified according to profession.

**Variables**	**Non-doctor/nurse**		**Doctor/nurse**		***p*-value**
	**(*****n*** **=** **945)**		**(*****n*** **=** **1,868)**		
**Metabolic syndrome**					
WC, cm (mean, SD)	82.24 (9.77)		75.32 (9.35)		<0.001
SBP, mmHg (mean, SD)	124.57 (18.21)		115.74 (13.44)		<0.001
DBP, mmHg (mean, SD)	73.21 (10.85)		66.35 (9.21)		<0.001
BS, mg/dL (mean, SD)	101.35 (20.18)		92.43 (14.71)		<0.001
TG, mg/dl (mean, SD)	116.43 (42.83)		85.31 (35.74)		<0.001
HDL-C, mg/dl (mean, SD)	57.75 (14.22)		62.47 (13.15)		<0.001
Metabolic syndrome, *n* (%)	314	29.81	281	13.54	<0.001
Central obesity, *n* (%) ^f^	423	40.14	590	28.38	<0.001
Elevated blood pressure, *n* (%) ^g^	405	38.36	440	21.17	<0.001
Hyperglycemia, *n* (%) ^h^	428	40.56	343	19.44	<0.001
Hypertriglyceridemia, *n* (%) ^i^	241	22.87	240	11.53	<0.001
Low HDL-C, *n* (%) ^j^	283	26.78	376	18.11	<0.001
**Maslach Burnout Inventory (MBI) (Mean, SD)**					
Burnout (*n*, %)	68(6.42)		139 (6.68)		0.88
Emotional exhaustion	22.74 (9.45)		27.14 (9.78)		<0.001
Personal accomplishment	24.51 (4.35)		27.88 (5.30)		<0.001
Depersonalization	8.78 (4.57)		12.42 (5.12)		<0.001
Emotional exhaustion, ≧27 (*n*, %)	280	26.54	744	35.78	<0.001
Personal accomplishment, ≦31 (*n*, %)	348	32.98	688	33.12	0.74
Depersonalization, ≧13 (*n*, %)	432	40.95	534	25.68	<0.001

f *Waist circumference ≥90 cm in men or ≥80 cm in women*.

g *SBP≧130mmHg or DBP≧85mmHg or self-reported hypertension*.

h *Fasting blood glucose ≥100 mg/dL or self-reported diabetes mellitus*.

i *TG≥150 mg/dL*.

j *HDL-C <40 mg/dL in men or <50 mg/dL in women*.

*n, numbers; SD, Standard deviation; WC, waist circumference; BP, blood pressure; BS, blood sugar; TG, triglyceride; HDL-C, high density lipoprotein-cholesterol*.

### Multivariate Logistic Regression Analysis of Burnout and Working Factors Influencing MetS Stratified According to Profession

After adjusting for age, sex, educational level, lifestyle characteristics, and sleep quality (Table 3), the results showed that burnout in the doctor/nurse group increased the risk of MetS by 1.96% (95% CI = 1.15–3.35; *p* = 0.01). In addition, the risk of MetS according to seniority was higher in participants working for >10 years (OR = 1.30; 95% CI = 1.02–2.90; *p* = 0.04); but lower participants working for seniority of 2–4 years (OR = 0.49; 95% CI = 0.26–0.99; *p* = 0.04). Night shift increased the risk of MetS in the doctor/nurse group (OR = 2.72; 95% CI = 1.39–5.34; *p* = 0.004).

**Table 3:** Multivariate logistic regression analysis of burnout and working factors influencing MetS stratified according to profession.

**Variables**	**Total**			**Non-doctor/nurse**			**Doctor/nurse**		
	**Odds ratio**	**95% CI**	***p*-value**	**Odds ratio**	**95% CI**	***p*-value**	**Odds ratio**	**95% CI**	***p*-value**
Burnout (yes vs. no)	1.50	1.02–2.31	0.04	1.11	0.54–2.27	0.78	1.96	1.15–3.35	0.01
**Seniority**									
<2 years	1	–	–	1	–	–	1	–	–
2–4 years	0.80	0.50–1.25	0.32	1.21	0.64–2.29	0.56	0.49	0.26–0.99	0.04
4–10 years	1.35	0.90–2.00	0.15	1.36	0.75–2.47	0.32	1.35	0.77–2.35	0.29
>10 yeras	1.42	0.89–2.24	0.14	1.83	0.97–3.34	0.06	1.30	1.02–2.90	0.04
**Working hours/week**									
≦45 h	1	–	–	1	–	–	1	–	–
46–50 h	1.20	0.89–1.61	0.23	1.04	0.66–1.65	0.86	1.27	0.86–1.88	0.24
51–59 h	1.50	0.85–2.64	0.16	2.09	0.53–8.31	0.29	1.44	0.75–2.76	0.27
≧60 h	0.71	0.34–1.51	0.38	1.51	0.20–11.41	0.69	0.73	0.31–1.76	0.49
**Work style**									
Regular class	1	–	–	1	–	–	1	–	–
Night shift	2.46	1.40–4.30	0.002	2.06	0.61–6.95	0.25	2.72	1.39–5.34	0.004
Three shift	1.05	0.79–1.40	0.72	0.54	0.63–1.44	0.82	1.14	0.74–1.76	0.56

*Adjusted by age, sex, educational level, and lifestyle characteristics (exercise, smoking, drink, fruit intake, vegetable intake), and sleep quality*.

Supplementary Table 1 showed the correlation of seniority with working hours/week and shift work by Spearman correlation coefficient matrix. The data showed below ±0.5 that means no or poor correlation.

### Analysis of the MetS Prevalence Stratified According to Age, Sex, and Profession

The results obtained after stratification according to age and sex suggested that female participants with burnout had a higher prevalence of MetS than female participants without burnout in the younger stratum (OR = 2.34; 95% CI = 1.14–4.80) (Table 4). When stratifying according to age and profession, doctor/nurse staff with burnout had a higher prevalence of MetS than the doctor/nurse staff without burnout in the younger cohort (OR= 2.32, 95% CI = 1.11–4.87) (Table 5). In male participants, older participants or non-doctor/nurse staff, the prevalence of MetS was not statistically significantly different among the burnout and non-burnout groups (Tables 4, 5).

**Table 4:** The association between burnout and prevalence of MetS by using logistic regression analysis stratified according to age and sex.

**Subgroup**	**No. of Mets**	**%**	**Model 1**			**Model 2**			**Model 3**			**Model 4**		
			**OR**	**95% CI**	***p*-value**	**OR**	**95% CI**	***p*-value**	**OR**	**95% CI**	***p*-value**	**OR**	**95% CI**	***p*-value**
**20–40 years and female (*****n*** **=** **1,541)**														
No-Burnout (1,454)	115	7.89	1	–	–	1	–	–	1	–	–	1	–	–
Burnout (87)	18	20.51	3.04	1.58–5.85	0.001	2.93	1.52–5.68	0.001	2.45	1.22–4.92	0.01	2.34	1.14–4.80	0.02
**20–40 years and male (*****n*** **=** **188)**														
No–Burnout (167)	22	13.33	1	–	–	1	–	–	1	–	–	1	–	–
Burnout (21)	4	21.11	1.62	0.41–6.39	0.49	1.65	0.41–6.56	0.48	2.37	0.53–10.58	0.26	2.15	0.38–12.03	0.38
**41–73 years and female (*****n*** **=** **1,260)**														
No–Burnout (1,170)	352	30.10	1	–	–	1	–	–	1	–	–	1	–	–
Burnout (90)	32	35.80	1.30	0.74–2.28	0.36	1.37	0.77–2.44	0.29	1.33	0.74–2.40	0.34	1.25	0.69–2.29	0.46
**41–73 years and male (*****n*** **=** **144)**														
No-Burnout (138)	61	44.35	1	–	–	1	–	–	1	–	–	1	–	–
Burnout (5)	1	20.00	0.23	0.03–2.13	0.20	0.19	0.02–1.84	0.15	0.24	0.02–2.82	0.26	0.28	0.02–3.59	0.33

*Model 1: Unadjusted*.

*Model 2: Adjusted for educational level*.

*Model 3: Adjusted for model 2 added seniority, working hours, and work style*.

*Model 4: Adjusted for model 3 added exercise, smoking, drink, fruit intake, vegetable intake, and sleep quality*.

**Table 5:** The association between burnout and prevalence of MetS by using logistic regression analysis stratified according to age and profession.

**Subgroup/profession**	**No. of Mets**	**%**	**Model 1**			**Model 2**			**Model 3**			**Model 4**		
			**OR**	**95% CI**	***p*-value**	**OR**	**95% CI**	***p*-value**	**OR**	**95% CI**	***p*-value**	**OR**	**95% CI**	***p*-value**
**20–40 years and non-doctor/nurse (*****n*** **=** **363)**														
No-Burnout (336)	41	12.25	1	–	–	1	–	–	1	–	–	1	–	–
Burnout (27)	7	25.00	2.83	0.85–9.48	0.09	2.52	0.73–8.70	0.14	1.96	0.49–7.79	0.34	1.86	0.39–8.85	0.43
**20–40 years and doctor/nurse (*****n*** **=** **1,367)**														
No-Burnout (1,287)	101	7.88	1	–	–	1	–	–	1	–	–	1	–	–
Burnout (80)	13	16.67	2.77	1.41–5.45	0.003	2.75	1.39–5.43	0.004	2.51	1.22–5.15	0.01	2.32	1.11–4.87	0.03
**41–73 years and non-doctor/nurse (*****n*** **=** **689)**														
No-Burnout (648)	252	38.83	1	–	–	1	–	–	1	–	–	1	–	–
Burnout (41)	15	37.84	0.85	0.38–1.88	0.68	0.88	0.39–1.97	0.75	0.89	0.39–2.05	0.79	0.87	0.37–2.03	0.75
**41–73 years and doctor/nurse (*****n*** **=** **714)**														
No-Burnout (658)	159	24.20	1	–	–	1	–	–	1	–	–	1	–	–
Burnout (56)	17	30.00	1.61	0.76–3.38	0.21	1.60	0.75–3.41	0.22	1.92	0.87–4.22	0.11	1.74	0.77–3.90	0.18

*Model 1: Unadjusted*.

*Model 2: Adjusted for educational level*.

*Model 3: Adjusted for model 2 added seniority, working hours, and work style*.

*Model 4: Adjusted for model 3 added exercise, smoking, drink, fruit intake, vegetable intake, and sleep quality*.

## Discussion

In our study, doctor/nurse staff with night shifts and seniority >10 years had a higher OR of MetS, but those with seniority of 2–4 years had a decreased OR of MetS. In a further stratified analysis, it was found that female participants aged 20–40 years had a higher OR of MetS after adjusting for the burnout factor than those in other age and sex groups. At the same time, our results showed that young doctor/nurse staff with burnout had a higher OR of MetS compared to older doctor/nurse staff and non-doctor/nurse staff. Therefore, burnout associated with an increasing OR of MetS among young female professionals and doctors/nurses.

Our results showed that overall prevalence of MetS in all HCWs was 19.0%, higher than reported in another previous study in a tertiary hospital in Taiwan (12%) (3). The prevalence of MetS in the non-doctor/ nurse and doctor/nurse groups was 29.81 and 13.54%, respectively; the prevalence of MetS in the former group was similar to that reported in the general population in a previous Taiwanese study (25.5% of men and 31.5% of women) (18), whereas that in the latter group was lower than that reported in the general population, but similar to that reported in study conducted by Yeh et al. (3). The main reason may be that doctor/nurse group included young female professionals with healthy worker effect (3). A study by Hwang et al. analyzed a sample of the general population and observed that women aged <50 years had a low prevalence of MetS than men in the same age group (19), which was attributed to the hypothesis of a persistent hormone effect in premenopausal women (20). In our study, the doctor/nurse group predominantly included female professionals aged <40 years (92.6% and 36.1 years) compared to the non-doctor/ nurse group (80.6% and 45.2 years), and the hypothesis observed in the study by Hwang et al. can corroborate to the understanding of the lower prevalence of MetS in these groups.

However, in the study by Wei et al., the prevalence of MetS was 12.1% in a sample of workers in Taiwan (21), which is in contrast to our findings that the prevalence of MetS in hospital doctor/nurse employees was not lower and that those in the non-doctor/nurse group had a higher prevalence of MetS that as the general population. Therefore, hospital HCWs have a higher risk of being diagnosed with MetS due to the work environment. Bergmann et al., Chandola et al., and Garbarino et al. observed that work stress, long hours of work, and burnout are associated with MetS (22–24). In our study, we found that the prevalence of burnout among was 6.6, 6.42, and 6.68%, respectively, which not statistically significantly different (*p* = 0.88). However, in a previous regional hospital study in Taiwan, a higher prevalence of work-related burnout was observed in nurses (66%) and physicians (38.6%) (2).

There are several possible reasons for the different in the prevalence of burnout. First, we used the Chinese version of the MBI-HSS (15, 16), which has good reliability and validity for hospital employees (Cronbach's α: 0.84), which was different from other studies that used the Chinese version of the Copenhagen Burnout Inventory (2). Second, in this study, participants were younger (median age 38.4 years) than those in other studies (4, 9). Finally, our study participants had a high level of education (higher than college: 97.9%), and a previous study reported that women with high burnout scores had a higher frequency of low levels of education (25).

A previous study showed that the prevalence and risk of MetS increases with age, which has a positive correlation with seniority (17). A study in Mexico on tertiary hospital nurses showed that working in the night shift and seniority ≥15 years were associated with adiposity-based chronic disease (26). In our study, we found that doctor/nurse staff on night shift and with seniority >10 years had a higher risk of MetS; these results were consistent with those of previous studies (26–28). The novel finding in our study was that seniority of 2–4 years had a decreased risk of MetS, the possible reason was younger age of these group.

Cross-sectional studies by Copertaro et al. and Ha et al. and retrospective studies by Biggi et al. and Brum et al. have suggested an association between shift work and MetS (29–32). In a prospective study with a 5-year follow-up with 387 female employees of a Taiwan plant, Lin et al. showed that rotating shift work significantly increased the risk of MetS (33). Coworkers who initially had one or two risk factors for metabolic disease were 4.6 and 12.7 times more likely to develop the condition after 5 years. In a 4-year prospective study by Pietroiusti et al., an association between burnout and night shifts was observed in health workers (hazard ratio: 5.10; 95% CI = 2.15–12.11; *p* = 0.001) (34), thus corroborating with the hypothesis that a high level of stress during night shift may have an influence on MetS (23, 35).

A study by Chou et al. conducted with a sample from a Taiwanese hospital revealed that being in the younger age group, working overtime; being a nurse or doctor, and having a high-tension job were associated with high burnout (2). The burnout rate of nurses was >50% (2). Wang et al. showed that doctors had a higher stress rate than the general population. Shanafelt et al. reported that doctors had a greater degree of suffering than individuals in other professions (37.9% vs. 27.8%, respectively) (36, 37). Several studies have described that chronic work stress and long working hours, which could lead to reduction in sleep and exercise, and changes in eating habits, can be highly associated with the prevalence of MetS (3, 24, 27, 28). A study conducted in Mexico on tertiary hospital nurses showed that unhealthy lifestyle (<3 days per week and/or <30 min per session of physical activity and poor dietary habits) was associated with adiposity-based chronic disease (26). In our study, higher working hours, night shifts, lower exercise, and less healthy habits were found in doctor/nurse group compare to non-doctor/nurse group.

In our study, we found no statistically significant difference in terms of sleep duration between the non-doctor/nurse and doctor/nurse groups (6.58 vs. 6.60 h, *p* = 0.325), and poor self-assessment sleep quality was higher in the doctor/nurse group than in the non-doctor/nurse group (*p* < 0.001). Previous studies extensively review the published literature and provide a comprehensive overview on sleep quality in MetS, the results showed the sleep quality played an important role to be as a modulator of metabolic homeostasis (38, 39). The sleeping quality was adjusted in our analysis to avoid interference with the results.

The relationship between burnout, night shift, and sleep condition and MetS can be grouped into three categories. (1) Hormone activity: chronic stress is associated with hyperstimulation of the hypothalamic-pituitary axis; as a consequence, several hormones participate in these reactions, such as growth hormone, thyroid hormone, insulin, ghrelin, leptin, and cortisol (17–20, 40, 41). (2) Inflammation activity: burnout and sleep insufficiency increase the level of pro-inflammatory cytokine (such as C-reactive protein) (42, 43). (3) Sympathetic activity: a stressful lifestyle and short sleep duration stimulate the sympathetic–adrenal–medullary axis, stimulating the adrenal glands to release catecholamines (e.g., epinephrine and norepinephrine) (23, 24).

The components that showed the highest prevalence of MetS were hyperglycemia (40.56%) and central obesity (40.14%) in the non-doctor/nurse group and central obesity (28.38%) and hypertension (21.17%) in the doctor/nurse group. Similar findings were reported by Yeh et al., Pietroiusti et al., and Basei Rossa et al., who corroborate the hypothesis that blood glucose control, WC, and BP are important to improve the health status of hospital employees (3, 34, 44).

A previous study reported that burnout level reduced with increasing age in men, but the association was bimodal in women, with women aged 20–35 and >55 years showing the highest burnout level (45). When associations are examined by sex, women appear to report higher levels of burnout than that in men (46). The reasons included work and non-work stressors.

In the early stages of their careers, younger workers need to master their job requirements and face a greater risk of burnout (47). In addition, younger employees may need to juggle work–family conflicts, which is another risk factor that leads to burnout (48). However, with successful work mastery, employees can adapt to work requirements and demands, and burnout may begin to decline with age. In addition, work-family conflicts may also be reduced (49), thereby reducing their impact on burnout.

Associations between age and burnout were strongly moderated by sex. Men and women have different levels of exposure to work and non-work stressors (50). Generally, men are often exposed to better working conditions and lower work-family conflicts than women (51). Men and women in the workplace also tend to respond differently to different work and non-work stressors (work–family conflict) (52, 53).

In a further stratified analysis, we found that female professionals aged 20–40 years had a lower prevalence of MetS from previous studies but had a higher risk of MetS after adjusting for the burnout factor than professionals in other ages and sex groups. According to the above findings, burnout, shift work, and poor sleep conditions induce MetS. Different ages and sex induce burnout, which can further lead to MetS. This fact suggests that acquiring work mastery (such as a doctor/nurse job) combined with work–family conflicts (as the principal care provider in the family) is often higher in women than in men (51, 52). In general, women are more stressed, leading to burnout, which in turn induces a higher risk for MetS.

An analysis of the association between burnout and MetS in hospital HCWs, between the different professions, and an analysis stratified by age, sex, and profession were the strong points of this study. A unique feature of this study is that we draw on previous MetS analysis and burnout prevalence in medical teams, including younger age [a significant factor in the burnout domains (7); higher for young female nursing staff [OR, 20–40 years vs. 41–65 years = 1.74 vs. 1.29 (2)] and profession [the greater the constancy and the intense the relationship with the patients, the greater the care needs to be provided, which leads to a greater risk of burnout (5)]. To our knowledge, this is a result that has not been specifically analyzed yet.

This study has some limitations. First, it was a single-institution study. Second, the causality of the associations was not analyzed because of the cross-sectional study design. Third, the study had a small sample size of participants from different departments, including: doctors 110 (3.5%), nurses 1968 (62.8%), medical technology department [radiology 52 (1.7%), laboratory 105 (3.4%), pathology 42 (1.3%), others 284 (9.1%)], nutrition department 64 (2.0%), pharmacy department 98 (3.1%), and administration 410 (13.1%). We cannot perform a more detailed departmental analysis. Fourth, the actual response rate was a little low (63.2% [2,078/3,288] in the doctor/nurse staff and 54.3% [1,055/1,943] in the non-doctor/nurse staff). Most of the HCWs felt tired and dull to answer the questionnaire. They did not fill out the questionnaire more than third/fourth. Fifth, sparse effects that may increase the probability of monotone likelihood in this study [OR in Table 3 (night shift) and 4 (burnout) seems inflated in this study] as well as a further limitation (54). Sixth, the cross-sectional studies had Neyman bias because it was confirmed that the questionnaire could not obtain whether the subjects had a burnout in the past or present. However, under such a selection bias, the results are still statistically different, which means that the results still have research value. Finally, there is a need to acknowledge the lack of supporting data on the levels of hormone and pro–inflammation cytokines to further strengthen the explanations of the mechanisms.

In the future, prospective studies involving multiple medical centers are needed to confirm the evidence of this research. In addition, we hope to use the occupational health education and training program to let HCWs understand that a lot of information can be obtained through questionnaire analysis, which will help us improve our policies. More details about working conditions, work–family conflicts, and biochemical biomarker can be added to accumulate more knowledge about the causal relationship and mechanism between work stress, burnout and MetS.

## Conclusions

A significant association between burnout and MetS was observed in young female professionals and young doctors/nurses. For the doctor/nurse staff with higher seniority or more night shifts, strategies are needed to prevent burnout and MetS. Agencies need to incorporate stress management into current guidelines to prevent the MetS, especially in the hospital workplace.

## Data Availability Statement

The original contributions presented in the study are included in the article/Supplementary Material, further inquiries can be directed to the corresponding author/s.

## Ethics Statement

The studies involving human participants were reviewed and approved by Human Research Ethics Committee at Mackay Memorial Hospital (project research number 18MMHISO150). The patients/participants provided their written informed consent to participate in this study.

## Author Contributions

M-TT participated in the design and conception of the study and its coordination, acquisition of data, carried out statistical analysis, and drafted the manuscript. H-JT participated in the conception and design of the study and reviewed the analysis and manuscript. All authors contributed to the article and approved the submitted version.

## Conflict of Interest

The authors declare that the research was conducted in the absence of any commercial or financial relationships that could be construed as a potential conflict of interest.

## Publisher's Note

All claims expressed in this article are solely those of the authors and do not necessarily represent those of their affiliated organizations, or those of the publisher, the editors and the reviewers. Any product that may be evaluated in this article, or claim that may be made by its manufacturer, is not guaranteed or endorsed by the publisher.
