# Supplementary material for: Age, Sex, and Profession Difference Among Health Care Workers With Burnout and Metabolic Syndrome in Taiwan Tertiary Hospital—A Cross-Section Study
Source: Front Med (Lausanne). 2022 Apr 14;9:854403. doi: 10.3389/fmed.2022.854403 (PMC9048413; doi:10.3389/fmed.2022.854403)
Supplement: Supplementary file 1 [file Table_1.DOCX]

Supplement table Spearman correlation coefficient matrix between seniority and working factors

|  | Seniority | Working hours/week | Work style |
| --- | --- | --- | --- |
| Seniority | 1.00 |  |  |
| Working hours/week | -0.114 ** | 1.00 |  |
| Work style | -0.260 ** | 0.158 ** | 1.00 |

** p < 0.001
